# Systems science for developing policy to improve physical activity, the Caribbean

**DOI:** 10.2471/BLT.20.285297

**Published:** 2021-08-13

**Authors:** Leonor Guariguata, Nigel Unwin, Leandro Garcia, James Woodcock, T Alafia Samuels, Cornelia Guell

**Affiliations:** a George Alleyne Chronic Disease Research Centre, University of the West Indies, Avalon Jemmott's Lane, Bridgetown, St Michael, BB11115, Barbados.; b School of Medicine, Dentistry and Biomedical Sciences, Queen’s University, Belfast, England.; c MRC Epidemiology Unit, University of Cambridge School of Clinical Medicine, Cambridge, England.; d Caribbean Institute for Health Research, University of the West Indies, Kingston, Jamaica.; e European Centre for Environment and Human Health, University of Exeter Medical School, Truro, England.

## Abstract

The World Health Organization (WHO) Global Action Plan on Physical Activity recommends adopting a systems approach to implementing and tailoring actions according to local contexts. We held group model-building workshops with key stakeholders in the Caribbean region to develop a causal loop diagram to describe the system driving the increasing physical inactivity in the region and envision the most effective ways of intervening in that system to encourage and promote physical activity. We used the causal loop diagram to inform how the WHO Global Action Plan on Physical Activity might be adapted to a local context. Although the WHO recommendations aligned well with our causal loop diagram, the diagram also illustrates the importance of local context in determining how interventions should be coordinated and implemented. Some interventions included creating safe physical activity spaces for both sexes, tackling negative attitudes to physical activity in certain contexts, including in schools and workplaces, and improving infrastructure for active transport. The causal loop diagram may also help understand how policies may be undermined or supported by key actors or where policies should be coordinated. We demonstrate how, in a region with a high level of physical inactivity and low resources, applying systems thinking with relevant stakeholders can help the targeted adaptation of global recommendations to local contexts.

## Résumé

Le Plan d'action mondial pour l'activité physique élaboré par l'Organisation mondiale de la Santé (OMS) recommande d'adopter une approche systémique pour la création et le déploiement d'actions adaptées aux contextes locaux. Nous avons organisé des ateliers de construction de modèles regroupant divers acteurs clés de la région Caraïbes. Objectif: développer un diagramme de boucles causales afin d'identifier le système à l'origine de la sédentarité croissante dans cette région, mais aussi de concevoir les moyens les plus efficaces pour s'immiscer dans ce système en vue d'encourager et de promouvoir l'exercice physique. Nous avons employé le diagramme de boucles causales pour définir comment le Plan d'action mondial pour l'activité physique de l'OMS peut être adapté au contexte local. Bien que les recommandations de l'OMS se rapprochent considérablement de notre diagramme, ce dernier illustre aussi l'importance du contexte local dans la manière dont les interventions sont censées être coordonnées et mises en œuvre. Certaines de ces interventions prévoyaient d'ouvrir des espaces sécurisés dédiés à la pratique sportive pour les deux sexes, de lutter contre les attitudes négatives vis-à-vis de l'activité physique dans des situations spécifiques, notamment à l'école et au travail, et de rendre les infrastructures compatibles avec les modes de transport actifs. Le diagramme de boucles causales permet en outre de mieux comprendre comment les acteurs clés peuvent soutenir ou au contraire discréditer les politiques en la matière, et de voir où ces politiques ont besoin de coordination. Nous démontrons comment, dans une région marquée par un taux de sédentarité élevé et de faibles ressources, l'adoption d'une approche systémique impliquant les principaux intervenants peut contribuer à ajuster avec précision des recommandations mondiales à des contextes locaux.

## Resumen

El Plan de acción mundial sobre actividad física de la Organización Mundial de la Salud (OMS) recomienda adoptar un enfoque sistémico para implementar y adaptar las acciones según los contextos locales. Celebramos talleres de construcción de modelos de grupo con las principales partes interesadas en la región del Caribe para desarrollar un diagrama de circuito causal para describir el sistema que impulsa la creciente inactividad física en la región y prever las formas más eficaces de intervenir en ese sistema para fomentar y promover la actividad física. Utilizamos el diagrama de circuito causal para informar sobre cómo se podría adaptar el Plan de acción mundial sobre actividad física de la OMS a un contexto local. Aunque las recomendaciones de la OMS se ajustaban bien a nuestro diagrama, este también ilustra la importancia del contexto local a la hora de determinar cómo deben coordinarse y aplicarse las intervenciones. Algunas intervenciones incluyen la creación de espacios seguros para la actividad física para ambos sexos, la lucha contra las actitudes negativas hacia la actividad física en determinados contextos, incluidos los colegios y los lugares de trabajo, y la mejora de las infraestructuras para el transporte activo. El diagrama de circuito causal también puede ayudar a entender cómo las políticas pueden ser socavadas o apoyadas por actores clave o dónde deben coordinarse las políticas. Demostramos cómo, en una región con un alto nivel de inactividad física y pocos recursos, la aplicación del pensamiento sistémico con las partes interesadas pertinentes puede ayudar a la adaptación específica de las recomendaciones globales a los contextos locales.

## ملخص

توصي خطة العمل العالمية لمنظمة الصحة العالمية (WHO) بشأن النشاط البدني، باعتماد نهج للنظم لتنفيذ الإجراءات وتخصيصها وفقًا للأوضاع المحلية. لقد عقدنا ورش عمل جماعية لبناء النماذج مع أصحاب المصلحة الأساسيين في منطقة البحر الكاريبي، وذلك لتطوير مخطط حلقي سببي لوصف النظام الذي يؤدي إلى زيادة الخمول البدني في المنطقة، والتكهن بأكثر الطرق فعالية للتدخل في هذا النظام لتشجيع النشاط البدني والارتقاء به. لقد استخدمنا المخطط الحلقي السببي للتعرف على كيفية تخصيص خطة العمل العالمية لمنظمة الصحة العالمية بشأن النشاط البدني لتناسب الوضع المحلي. على الرغم من أن توصيات منظمة الصحة العالمية تتسق تمامًا مع المخطط الحلقي السببي لدينا، إلا أن هذا المخطط يوضح أيضًا أهمية الوضع المحلي في تحديد كيف يجب تنسيق التدخلات وتنفيذها. تضمنت بعض التدخلات إنشاء مساحات آمنة للنشاط البدني لكلا الجنسين، ومعالجة المواقف السلبية تجاه النشاط البدني في أوضاع معينة، بما في ذلك في المدارس وأماكن العمل، وتحسين البنية التحتية للنقل النشط. قد يساعد المخطط الحلقي السببي أيضًا في فهم كيفية تقويض السياسات أو دعمها من جانب الجهات الفاعلة الرئيسية، أو أين ينبغي التنسيق بين السياسات. نحن نوضح في منطقة ما ذات مستوى عالٍ من الخمول البدني والموارد المنخفضة، كيف يمكن لتطبيق التفكير النظامي مع أصحاب المصلحة ذوي الصلة، أن يساعد في تحقيق التكيف المستهدف للتوصيات العالمية لتناسب الأوضاع المحلية.

## 摘要

世界卫生组织 (WHO) 身体活动全球行动计划建议采用系统方法,根据当地情况实施和调整行动。我们与加勒比地区的主要利益相关者举行了小组模型构建研讨会,制定因果环路图来描述导致该地区身体活动不足加剧的系统,并构思对该系统最有效的干预方式以鼓励和促进身体活动。我们使用因果环路图来了解世卫组织身体活动全球行动计划可如何适应当地情况。尽管世卫组织的建议与我们的因果环路图非常吻合,但该图还说明了当地情况在决定如何协调和实施干预措施方面起到的重要作用。一些干预措施包括为男女双方创造安全的身体活动空间,解决人们在某些环境下(包括在学校和工作场所)对身体活动持有的消极态度,以及改善主动交通方式的配套基础设施建设。因果环路图也有助于了解主要参与者如何削弱或支持这些政策,或者应该在哪些地方调整这些政策。我们展示了在一个资源匮乏、身体活动高度不足的地区,如何与相关利益相关者共同运用系统思维,有针对性地调整全球建议以适应当地情况。

## Резюме

Глобальный план действий Всемирной организации здравоохранения (ВОЗ) по повышению уровня физической активности рекомендует применять системный подход к реализации и адаптации действий с учетом местных условий. Авторы провели групповые семинары по построению моделей с основными заинтересованными сторонами в регионе Карибского бассейна, чтобы разработать диаграмму причинно-следственной связи для описания системы, способствующей повышению уровня физической активности в регионе, а также представить наиболее эффективные способы работы данной системы для стимулирования и поощрения физической активности. Диаграмма причинно-следственной связи использовалась для того, чтобы определить, каким образом Глобальный план действий ВОЗ по повышению уровня физической активности можно адаптировать к местным условиям. Хотя рекомендации ВОЗ надлежащим образом согласуются с нашей диаграммой причинно-следственной связи, она также демонстрирует важность местных условий в определении того, каким образом мероприятия должны координироваться и осуществляться. Некоторые мероприятия включали создание безопасных мест для физической активности для представителей обоих полов, борьбу с негативным отношением к физической активности в определенных условиях, в том числе в школах и на рабочих местах, а также улучшение инфраструктуры для активных видов транспорта. Диаграмма причинно-следственной связи также может помочь понять, как ключевые участники процесса могут подорвать или поддержать политику или где следует координировать политику. Было показано, как в регионе с низким уровнем физической активности и ограниченными ресурсами применение системного мышления в работе с соответствующими заинтересованными сторонами может помочь целенаправленной адаптации глобальных рекомендаций к местным условиям.

## Introduction

Physical inactivity is a major driver of poor health globally and a risk factor for noncommunicable diseases.^1^ About a third of adults in the Caribbean are estimated to be physically inactive^2^ with rates of physical activity static or declining since 2001.^3^^–^^5^ Caribbean women are more than twice as physically inactive on average than men^2^ and 85% of adolescents are estimated to be physically inactive.^6^

In 2018, the World Health Organization (WHO) proposed the Global Action Plan on Physical Activity^7^ outlining interconnected areas for intervention across different levels of society (e.g. individual, community, subnational and national). The plan calls for a systems approach to reverse physical inactivity trends in recognition of the complex drivers and consequences affecting interventions.^8^ Several different approaches fall under the concept of systems thinking.^9^ One method presented here is group model building.^10^^,^^11^ The purpose of group model building is to capture and synthesize the different so-called mental models of each stakeholder and present these as a co-developed causal loop diagram with an emphasis on identifying feedback loops and causal pathways. The causal loop diagram may form the basis for a mathematical simulation model, although the diagram itself is considered a valuable output to conceptualize complex problems and can be useful in identifying intervention points and potential pathways of impact. Co-developing such diagrams can also be a way to engage stakeholders in systems thinking.^12^

Engaging directly with stakeholders using participatory approaches is important in setting agendas,^13^ developing policy-relevant actions^14^ and integrating knowledge into public health policy.^15^ Although not required, stakeholder engagement in building causal loop diagrams is an effective and valuable way to generate context-specific maps that capture the reality of people’s lives. Stakeholder engagement has been used in building causal loop diagrams to respond to noncommunicable diseases and their risk factors including active travel,^16^ obesity^17^ and unhealthy diet,^18^^,^^19^ and applied in other Small Island Developing States similar to the case presented here.^19^^,^^20^

The WHO Global Action Plan on Physical Activity recommends a systems approach to adapting interventions to the local context. However, guidance for implementation is not given.^7^ In this paper, we present one method for engaging key stakeholders in systems thinking on physical activity through group model building and the development of a causal loop diagram to identify areas for intervention. We examined the extent to which local priorities identified by stakeholders aligned with the recommendations of the WHO global action plan.

## Group model building

We invited key stakeholders in the Caribbean region to participate in a 2-day group model-building session in December 2016 that explored the determinants of obesity with a focus on physical inactivity and unhealthy diet.^21^ Box 1 gives a summary of the schedule of a typical group model-building workshop, although there is considerable flexibility in how these workshops can be conducted; for example, the number of stakeholders and sessions, in-person or virtual.^10^^,^^11^

Box 1 **Summary of the activities in the group model-building workshop**

**Day one**

**Introduction**

We welcomed the participants and gave a presentation describing previous work on noncommunicable diseases and the epidemiological profile in the Caribbean. We then presented the objectives of the workshop.

**Hopes and fears**

Participants wrote one hope and one fear for the workshop. We discussed these hopes and fears and displayed them on a wall.

**Core problem and introduction to feedback loops**

With the participants, we agreed to focus on a key core problem: declining physical activity in the region. Participants drew trends over time with projections of what would happen without any interventions (e.g. reference mode) and what the goal of interventions would be. We showed how causal loop diagrams are drawn using cars, road building and increasing traffic as an example.

**Nominal group technique**

We asked participants to list as many causes and consequences of low physical activity as they could. We collected, discussed and refined their views together and then grouped them into three themes.

**Causal loop diagrams**

We divided participants into three groups of five to six participants and each group took a theme to draw causal loop diagrams and how they related to low physical activity. We presented these diagrams to the whole group for discussion and refining.

**Day two**

**Places to intervene**

In small groups, participants used the causal loop diagrams to determine a list of places to intervene and possible policies that could move physical activity in the desired direction. We presented these diagrams to the whole group for discussion. Participants voted on the places to intervene that they thought would be most effective in increasing physical activity (each participant had three votes to place wherever they wanted). Together, we drew up a priority list of interventions.

**End reflections**

We revisited the causal loop diagrams, the policy levers and places to intervene, and the hopes and fears the participants recorded at the beginning of the workshop.

For this project, we conducted individual in-depth interviews with 15 key stakeholders before the workshop. We selected stakeholders purposefully from existing collaborations and networks with the University of the West Indies and local Caribbean government and civil society partners. Stakeholders included academics, health-care providers, government personnel, and staff from intergovernmental organizations and civil society from Barbados, Belize, Jamaica, and Saint Vincent and the Grenadines. In addition, we reviewed qualitative data from 72 interviews conducted in a seven-country case study evaluating the 2007 Port-of-Spain Declaration on Noncommunicable Diseases.^22^ Guidelines for group model building recommend a limit of 15 participants;^10^ of the 15 stakeholders invited, 12 participated in the 2-day workshop. We synthesized the findings from the interviews and presented them to the group of stakeholders at the beginning of the workshop as a way to establish a baseline for discussion.

Using established methods for group model building,^23^^,^^24^ we asked stakeholders to identify the common causes, drivers and consequences of low physical activity across the Caribbean. Stakeholders grouped these variables into three themes: social and cultural influences; the environment that promotes physical activity; and exercise and sports. We divided stakeholders into working groups to draw causal loop diagrams on each of those themes. We then transcribed causal loop diagrams from paper versions to digital copies using the simulation software Vensim PLE (Ventana Systems, Inc., Harvard, United States of America).^25^

**Fig. 1:**
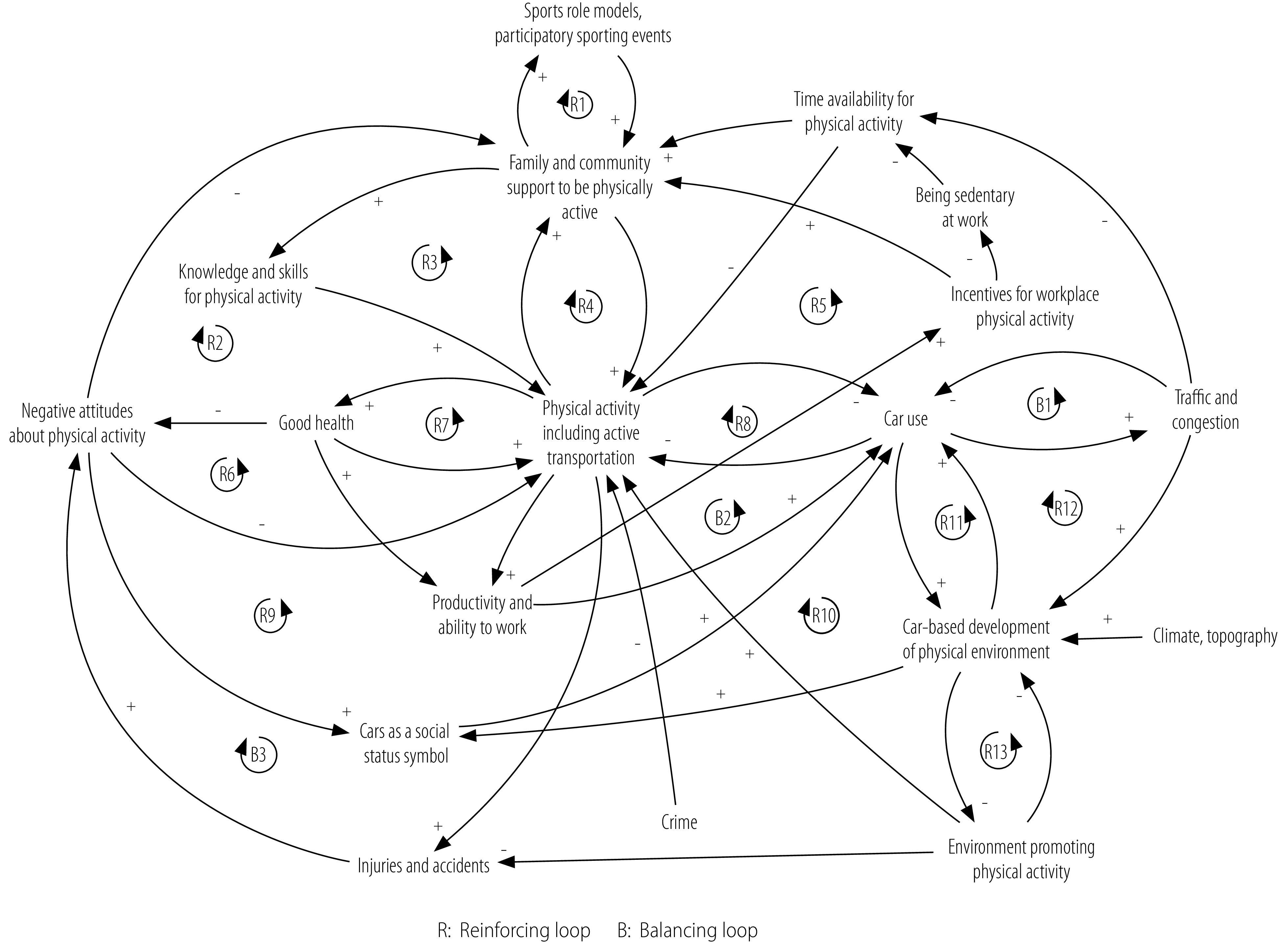
Summary causal loop diagram of the stakeholder developed system driving physical activity in the Caribbean

Note: Arrows in the causal maps connect causal relationships; the direction of the arrow marks the causal direction. The plus or minus sign next to the arrow indicates the type of relationship. A plus sign marks a relationship where the two elements move in the same direction. A negative sign indicates movement in the opposite direction. The connections of these elements form loops. A reinforcing loop (R) leads to an increase or decrease over time and a balancing loop (B) leads to a shift back towards an equilibrium value. These loops act together to affect the parameters within a model, limiting and promoting growth and contraction and affecting the system’s overall behaviour over time.

We used these three causal loop diagrams in discussions with the stakeholders to think through important causal pathways and to envision and expand on interventions to increase physical activity and the potential consequences of such interventions. We then used the three causal loop diagrams from the workshop together with the qualitative evidence from interviews and the review of data from the seven-country study^22^ to construct a summary causal loop diagram (Fig. 1). Finally, we compared the summary causal loop diagram from the 2016 workshop with the 2018 recommendations in the WHO global action plan (Box 2) to examine how the causal loop diagram could be used to help align global recommended actions to local realities. 

Box 2 **Recommended policy actions in the WHO Global Action Plan on Physical Activity**

**Objective 1: create active societies**

Create a paradigm shift in all of society by enhancing knowledge and understanding of, and appreciation for, the multiple benefits of regular physical activity, according to ability and at all ages.

1.1 Implement best practice communication campaigns to increase knowledge and understanding of the benefits of physical activity.

1.2 Conduct national and community-based campaigns to improve awareness and understanding of social, economic and environmental co-benefits of physical activity, particularly alternative types of mobility which align with the sustainable development goals.

1.3 Implement regular mass participation initiatives in public spaces, engaging communities in free, enjoyable and affordable physical activity experiences.

1.4 Strengthen training of professionals in the health sector to increase knowledge and skills to contribute to a more active society.

**Objective 2: create active environments**

Create and maintain environments that promote and safeguard the rights of all people, of all ages, to have equitable access to safe places and spaces, in their cities and communities, in which to engage in regular physical activity, according to ability.

2.1 Strengthen the integration of urban and transport planning policies to prioritize compact, mixed-land use at all levels of government to promote walking, cycling, and other forms of mobility including the use of public transport.

2.2 Improve the level of service provided by walking and cycling network infrastructures to enable and promote active transport and public transport with safe and equitable access for all.

2.3 Accelerate implementation of policies to improve road safety and the safety of pedestrians, cyclists and other forms of mobility and public transport passengers, reducing risk for the most vulnerable road users.

2.4 Strengthen access to good-quality, safe, equitable and accessible public and green open spaces, networks, and recreational spaces (including river and coastal areas) and sports amenities by all people and in all communities.

2.5 Strengthen policy, regulatory and design guidelines and frameworks at national and subnational levels to promote spaces that are designed to enable people to be physically active and prioritize universal access to those spaces by pedestrians, cyclists and public transport.

**Objective 3: create active people**

Create and promote access to opportunities and programmes, across multiple settings, to help people of all ages and abilities to engage in regular physical activity as individuals, families and communities.

3.1 Strengthen provision of good-quality physical education and more positive experiences and opportunities for active recreation, sports and play for girls and boys, applying a whole-of-school approach across all levels of schooling to promote physical activity.

3.2 Implement and strengthen systems of patient assessment and counselling on increasing physical activity and reducing sedentary behaviour through primary and secondary health care and social services, ensuring community and patient involvement.

3.3 Enhance provision of, and opportunities for, more physical activity programmes and promotion in parks and other natural environments as well as in private and public workplaces, community centres, recreation and sport facilities and faith-based centres to support physical activity for all.

3.4 Enhance the provision of, and opportunities for, appropriately tailored programmes and services aimed at increasing physical activity and reducing sedentary behaviour in older adults to support healthy ageing.

3.5 Strengthen the development and implementation of programmes and services across various community settings to increase the opportunities for physical activity in the least active groups.

3.6 Implement whole-of-community initiatives that stimulate engagement by all stakeholders and optimize a combination of policy approaches, across different settings, to promote increased participation in physical activity, focusing on grassroots community engagement, co-development and ownership.

**Objective 4: create active systems**

Create and strengthen leadership, governance, multisectoral partnerships, workforce capabilities, advocacy and information systems across sectors to achieve excellence in resource mobilization and implementation of coordinated international, national and subnational action to increase physical activity and reduce sedentary behaviour.

4.1 Strengthen policy frameworks, leadership and governance systems to support implementation of actions aimed at increasing physical activity and reducing sedentary behaviour, including: multisectoral engagement and coordination mechanisms; policy coherence across sectors; guidelines, recommendations and action plans on physical activity and sedentary behaviour for all ages; and monitoring and evaluation of progress to strengthen accountability.

4.2 Enhance data systems and capabilities at the national and, where appropriate, subnational levels, to support regular population surveillance of physical activity and sedentary behaviour.

4.3 Strengthen national and institutional research and evaluation capacity and stimulate the application of digital technologies and innovation to accelerate the development and implementation of effective policy solutions.

4.4 Increase advocacy efforts to improve awareness and knowledge of, and engagement in, joint action at the global, regional and national levels, targeting key audiences and the wider community.

4.5 Strengthen financing mechanisms to secure sustained implementation of national and subnational action and the development of enabling systems that support the development and implementation of policies aimed at increasing physical activity and reducing sedentary behaviour.

Source: WHO Global Action Plan on Physical Activity 2018–2030.^7^

## Aligning local priorities

One of the main objectives of a group model-building session is to use a causal loop diagram to identify opportunities to intervene that can make use of feedback loops and improve the chances of success for policy interventions. Stakeholders described local causal pathways and identified intervention points of importance to the Caribbean region and how pathways act to increase or inhibit the effectiveness of interventions. We grouped these intervention points by the high-level themes in the WHO action plan and discussed and explored their alignment with the recommendations of the action plan.

### Create active societies

The WHO action plan proposes four policy actions to motivate a paradigm shift in all of society by enhancing knowledge and understanding of the multiple benefits of regular physical activity (Box 2).^7^

#### Local causal pathways

Stakeholders described some communities in the Caribbean as not supporting physical activity; they considered negative attitudes to physical activity one of the greatest threats to implementing effective interventions. These attitudes were in part a result of complex cultural norms that discourage sweating and associate physical activity (including active transport, e.g. walking or cycling for travel) with low socioeconomic status, and these norms may be difficult to change (Fig. 1; reinforcing feedback loop R6). However, the stakeholders described several pathways in the causal loop diagram that could work to reinforce social systems supporting physical activity. Strengthening of knowledge and skills through school- and community-based interventions could take advantage of a reinforcing feedback loop that reduces negative attitudes and creates a more supportive society (R3).

Stakeholders also described trends recognizable in other countries undergoing economic transitions – including a shift towards sedentary occupations, more time spent away from the household and more single-parent households – that were perceived to limit leisure-time physical activity.

#### Interventions

In the Caribbean, stakeholders thought that large participatory events, such as increasingly popular walking and/or running events, were a way to encourage a supportive community at all levels; from small community-based initiatives in public spaces (alignment with WHO action plan, Action 1.3; Box 2 and R1) to the development of physical education in schools (Action 3.1 and R4), and countrywide mass communication campaigns (Action 1.2 and R3). Stakeholders suggested engaging regional athletes to serve as champions of physical activity (R1). However, stakeholders cautioned that the sponsorship of some athletes by multinational food and drink corporations conflicts with the objective of lowering obesity. Stakeholders also proposed providing incentives for physical activity at the workplace as a way to reduce sedentary time, although they noted that this intervention was unlikely to be enough to compensate for the loss of occupational physical activity.

### Create active environments

The WHO action plan proposes five actions to create active environments (Box 2).^7^ These actions include supportive spaces that promote and safeguard the rights of all people to have equitable access to safe spaces where they can engage in regular physical activity.

#### Local causal pathways

Stakeholders noted that many Caribbean countries have a lot of physical space (Action 2.4) for leisure-time physical activity and active travel, but these spaces are poorly maintained or not always accessible to the general public (R13), and they are often unsafe. Furthermore, the humid tropical climate makes engaging in physical activity difficult. The physical environment in the Caribbean currently supports the use of motor vehicles and is continuing to develop in a reinforcing loop that competes directly with an environment supportive of physical activity (R8 and R11). This trend will increase traffic and congestion which may trigger a possible balancing loop (B1), but often public planning promotes the expansion of roads to accommodate cars, further reinforcing car use.

Stakeholders described an inconsistent pattern of development of spaces for physical activity. They considered some efforts made to develop well maintained and guarded spaces for physical activity (such as Emancipation Park in Kingston, Jamaica,^26^ or the boardwalk in Barbados)^27^ were positive infrastructure projects. However, they thought that such well-developed spaces are often not integrated into communities, especially communities with a lower socioeconomic status.^28^ Furthermore, many spaces would require travel to reach them, potentially widening inequalities. In addition, the development of spaces is often focused on use by tourists and not the local population.

#### Interventions

Stakeholders supported the development of walking and cycling infrastructure (Action 2.2). However, they thought that negative social perceptions of active transport could hinder infrastructure changes and hence there would be little public support for those changes (R9). Actions to reduce dependence on cars could have far-reaching effects, triggering several feedback loops that would directly influence physical activity (R8). Stakeholders also thought that reducing street crime would contribute substantially to reducing negative attitudes about active travel and leisure-time physical activity, a problem that is especially acute in urban settings (Action 2.3, R2 and R6).

One intervention proposed by stakeholders that is not directly called for in the WHO action plan is the use of financial interventions to create disincentives for car use. Stakeholders discussed taxing private cars within urban zones and investing in a safe and accessible public transportation system (Action 2.2).

### Create active people

Six policy actions in the WHO action plan outline multiple ways in which an increase in programmes and opportunities can help people engage in regular physical activity (Box 2).^7^

#### Local causal pathways

Stakeholders described time constraints and sedentary leisure time as undermining community-level physical activity and driving a loss of knowledge and skills (R3). They emphasized the difference in physical activity between men and women in the Caribbean – women are on average almost three times less physically active than men – and they described several contributing pathways.^29^ These pathways included: having more responsibilities in the house in addition to working, creating less leisure time for physical activity (R5); being discouraged from participating in physical activity because of negative attitudes to sweating (R2 and R4); being on average more obese than men and thus less physically able to engage in physical activity (R7); and having less access to spaces for leisure-time physical activity, especially sports, which are seen as more appropriate or safer for men.

#### Interventions

Stakeholders considered the support of families and communities through grassroots efforts a key area for intervention (Action 3.5). Regardless, interventions in all areas would have to give special attention to reducing barriers to women participating in physical activity and improving their physical activity (Action 3.6). Stakeholders again saw a role for large participatory events to help increase leisure-time physical activity (Action 1.3 and R1), especially if these target women. In addition, improving physical education in schools could improve community support for physical activity (Action 3.1), although this approach could take many years to show a change in physical activity levels (R2, R3 and R4). Furthermore, stakeholders saw a greater role for health-care providers and organizations focused on care for noncommunicable diseases to take the lead on strengthening skills for physical activity (Action 3.2).

### Create active systems

The WHO action plan outlines five actions to support the investments needed to strengthen implementation systems at national and subnational levels (Box 2).^7^

#### Local causal pathways

Many of the loops in the causal loop diagram are nested within each other, involve multiple sectors and have many connections to just a few variables. This pattern suggests that acting on those variables using multipronged and multilevel coordinated interventions could produce synergistic reinforcing loops.

#### Interventions

Stakeholders called for better policy coordination and a multisectoral approach to health that extends not just at the national level but also across the region (Action 4.1). To enact some of the policies discussed in the workshop, various government ministries, the private sector, nongovernmental organizations and intergovernmental agencies would need to cooperate towards a common goal over decades. Many of the islands and territories in the Caribbean are small and may lack the necessary resources to implement some of the discussed interventions by themselves. While the WHO action plan calls for strengthening national policy frameworks (Action 4.1), the stakeholders thought that support to regional groups that can implement measures across borders was especially important. Other areas recommended in the WHO action plan on actions for systems (e.g. financing mechanisms, information systems and research capacity) did not come up in the discussions.

## Coordinating policy

The system determining physical activity patterns that our stakeholders mapped reaches across sectors and acts at all levels. The patterns also align well with the recommendations of the WHO action plan on physical activity. Many of the themes and causal pathways explored in the Caribbean causal loop diagrams are also described in the systems framework supporting the development of the WHO action plan.^8^ Our summary causal loop diagram (Fig. 1) extends that framework by assigning causal direction and capturing feedback loops relevant to physical activity in the region. Engaging directly with stakeholders in the development of the causal loop diagram not only provided important local knowledge, but also helped the stakeholders to develop a broader, more systemic view of the determinants of physical activity. Ideally, the stakeholders participating in group model building will also be those who are empowered to enact or influence policies or interventions. In a setting such as the Caribbean, one person may hold many roles in government, academia, nongovernmental organizations and the community. Engaging with these stakeholders may provide the best chance of ensuring that the benefits of the systems approach presented here can be fully implemented.

Some multipronged, coordinated campaigns and projects focused on reducing obesity in the Caribbean, including through increasing physical activity, have taken place recently. The Jamaica Moves campaign^30^ that began in 2016 combines multimedia public health messaging and advertising with physical activity events, coproduction with community action groups and a corporate challenge component to engage the private sector. We are not aware of a formal evaluation of the campaign but its popularity has led to it being developed by the Caribbean Public Health Agency as the Caribbean Moves project to help other countries implement similar programmes.^31^

## Threats

In addition to outlining interventions and synergies between policies, the stakeholders discussed the threats and challenges to their development and implementation. These issues included: a lack of resources to change and maintain an environment and infrastructure that promotes physical activity; competing policy priorities (e.g. emerging infectious diseases and economic interests conflicting with obesity prevention) that could undermine efforts to coordinate across sectors; and the social, political and economic influence of commercial interests that are harmful to health and the environment, including car manufacturers, the fossil fuel industry and multinational food and drink corporations. These industries were seen as actively working to undermine physical activity in the region. Stakeholders also viewed the tourism sector as a potential threat where governments prioritize access for tourists to green and blue spaces (particularly coastlines) to the detriment of the local population. However, they emphasized that the tourism industry could be a partner in developing and maintaining environments for physical activity, but that governments must protect equitable access for the local people consistently across the region. The stakeholders also considered that a regional coordination mechanism to support best practice, share resources and create greater political authority for regulating harmful commercial and private interests would improve physical activity across the Caribbean.

## Challenges

Many methods exist for engaging in systems thinking for policy development; group model building is just one approach with a long history in systems dynamics.^10^ However, challenges exist to applying a systems approach. The outcomes of a group model-building session are sensitive to who participates in the sessions. It is important, then, to choose stakeholders carefully so that the group has knowledge of different aspects of a system and can provide a breadth of experience needed to draw a systems-wide causal loop diagram. In addition, mapping is ideally an iterative process and should be revisited and revised as more information becomes available. Finally, the group model-building process presented here can be used as a tool to guide priority-setting and the development of policy plans as well as for identifying information gaps and ways to fill those gaps. However, as a qualitative tool it cannot indicate potential effect sizes of interventions, nor the resources required to implement interventions. Mathematical simulation models, if data are available, can often be more useful in that regard. However, policy-makers in settings with limited data can still benefit from engaging in systems thinking methods that do not necessarily rely on simulation models. Group model building can help improve communication between stakeholders, develop commitment to a common cause, build consensus and motivate change.^12^^,^^32^

A systems approach to policy development requires policy-makers to look beyond one aspect of a complex problem. Considering a broader view of the drivers and outcomes around a problem can help mitigate unintended consequences and plan for innovative interventions that engage a wide range of actors.^9^ In the example presented here, the causal loop diagram focuses on improving physical activity in a region with the goal of reducing obesity and noncommunicable diseases. Thus, the drivers, determinants and outcomes of the wider system that leads to more obesity must also be considered if a true systems approach is to be adopted.
